# Hydrolytic Stability of Crosslinked, Highly Alkaline Diallyldimethylammonium Hydroxide Hydrogels

**DOI:** 10.3390/gels8100669

**Published:** 2022-10-19

**Authors:** Tim B. Mrohs, Oliver Weichold

**Affiliations:** Institute of Building Materials Research, RWTH Aachen University, Schinkelstraße 3, 52062 Aachen, Germany

**Keywords:** hydrogel, copolymer, durability, hydrolysis, swelling, rheology, crosslinker

## Abstract

The aim of this study was to evaluate the persistence of alkaline hydrogels based on a common (*N*,*N*′-methylenebisacrylamide, BIS) and three recently published tetraallyl crosslinkers. Such hydrogels have been shown to be suitable materials for the rehabilitation of cementitious materials. Of the four crosslinkers under investigation, *N*,*N*,*N*′,*N*′-tetraallylpiperazinium dibromide decomposed quickly in 1 m KOH solution and was not considered further. BIS showed the first signs of a decomposition after several days, while tetraallylammonium bromide and *N*,*N*,*N*′,*N*′-tetraallyltrimethylene dipiperidine dibromide remained unaffected. In contrast to BIS, which suffers from low solubility in water, the two tetraallyl crosslinkers show unlimited miscibility with diallyldimethylammonium hydroxide solutions. For the study, gels with up to 50 wt % crosslinker were prepared. Of these, gels containing tetraallylammonium bromide always show the highest degrees of swelling, with a peak value of 397 g/g at a content of 2 wt %. Under accelerated ageing at 60 °C for 28 d, gels crosslinked with BIS ultimately turned liquid, while the storage modulus and the degree of swelling of the two tetraallyl-crosslinked gels remained unchanged. This indicates that alkaline gels can be suitable for long application periods, which are common for rehabilitation measures in the construction industry.

## 1. Introduction

Hydrogels have become increasingly important in research and industry in recent years. Gels in general are crosslinked polymer networks, which can absorb and release various liquids without losing their discrete three-dimensional structure. If the absorbed medium is water, the polymer network is referred to as hydrogel [1]. The major advantage of hydrogels is the potential to specifically tailor the chemical structure, which allows for a wide range of applications. Non-ionic hydrogels, for example, are often used in protein analysis [2] or biomedical applications [3] due to their pH-independent swelling properties and their insensitivity to salt concentrations [4]. However, the majority of hydrogels are ionic, such as the well-known poly(sodium acrylate), which is used as a superabsorber for diapers [5] or as a shrinkage-reducing agent in concrete [6]. Ionic hydrogels usually exhibit significantly higher degrees of swelling and can respond to changes in pH value and/or salt concentration in the surrounding medium [7].

Hydrogels that are particularly suitable for the construction industry can be obtained by using cationic networks with hydroxide as a counterion; these have recently been realized based on diallyldimethylammonium hydroxide (DADMAOH) as a monomer and *N*,*N*′-methylenebisacrylamide (BIS) as a crosslinker. Such highly alkaline polymer networks were not only shown to be valuable materials in the rehabilitation of steel-reinforced concrete by exchanging carbonate ions in aged concrete with hydroxide ions, thereby restoring the high pH value necessary for preventing steel corrosion [8], but also as a coupling material for electrochemical chloride extraction [9]. A similar hydrogel was recently used to seal water-bearing cracks, while at the same time restoring the protective passive-layer on the exposed parts of steel rebars [10]. Although the system has already been tested successfully in field trials [9], the crosslinker BIS appears to be a weak point for a number of reasons: BIS suffers from a rather low solubility in water (approx. 20 g/L at 20 °C [11]), which limits the possibility to prepare firm gels. Moreover, as an acrylate derivative, BIS polymerizes significantly faster than the diallyl (DADMA^+^) unit, which was shown to lead to inhomogeneous networks [12]. Gels crosslinked with BIS also made the qualitative impression of softening over the course of several months. The hydrolysis of the bisamide liberates formaldehyde, which is undesirable in large-scale applications [13].

We have recently reported on the synthesis of three new tetraallylammonium-based crosslinkers, namely tetraallylammonium bromide, *N*,*N*,*N*′,*N*′-tetraallylpiperazinium dibromide, and *N*,*N*,*N*′,*N*′-tetraallyltrimethylene dipiperidine dibromide [12], and we used these to successfully crosslink diallyldimethylammonium chloride, a pH-neutral derivative of DADMAOH. Due to their better solubility in water, a wider range of crosslinking densities can be obtained, and due to their structural similarity, the copolymerisation leads to homogeneous networks [12]. The question now arises as to how these crosslinkers perform in the alkaline diallyldimethylammonium hydroxide (DADMAOH) system designed for application in, e.g., cementitious materials. This question is addressed by first evaluating the persistence of the pure compounds in alkaline media and then validating these findings by monitoring the rheological and swelling properties of gels under accelerated ageing. The results are compared to gels crosslinked with BIS.

## 2. Results and Discussion

**Figure 1:**
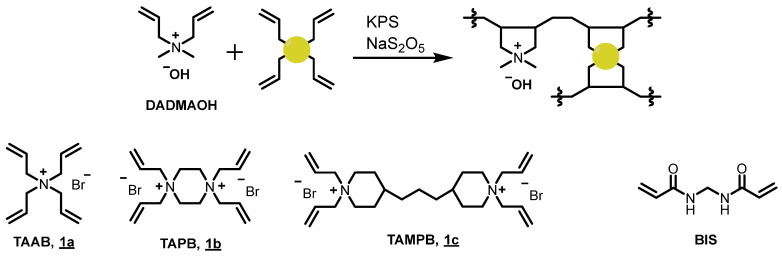
Top: general scheme for the crosslinking polymerization of DADMAOH; bottom: structures of the cross-linkers **1a**–**c** and BIS used in this study.

The copolymerization of DADMAOH with the crosslinkers **1a–c** follows the published procedure for the polymerization of DADMAOH with BIS using a redox initiation system consisting of potassium peroxodisulfate and sodium disulfite [8]. As shown in Figure 1, the diallyldimethylammonium unit polymerizes under ring closure, triggered by the attack of a radical. The same mechanism also operates in the crosslinkers **1a–c**. BIS, on the other hand, polymerises by linear radical addition to each of the double bonds—i.e., it does not form a ring—and, therefore, exhibits clearly different copolymerization characteristics than **1a–c** with DADMA^+^ monomers [12]. 

**Figure 2:**
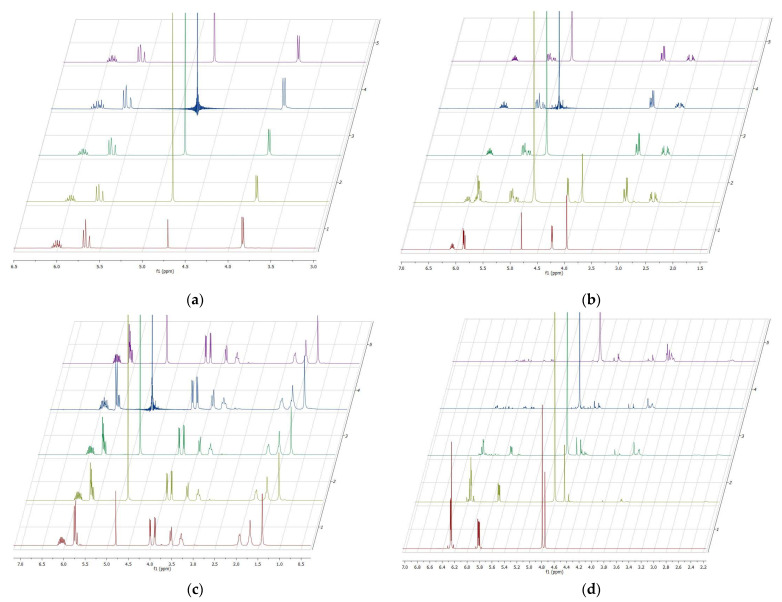
^1^H-NMR spectra of the pure crosslinkers in 1 m KOH solution in D_2_O over the course of 7 days: (**a**) tetraallyl ammonium bromide, (**b**) *N*,*N*,*N*′,*N*′-tetraallyl piperazinium dibromide, (**c**) *N*,*N*,*N*′,*N*′-tetraallyl trimethylene dipiperidine dibromide, (**d**) *BIS*. The numbers on the right axis indicate the time code: (1) reference spectrum without KOH, (2) 1 m KOH in D_2_O on the same day, (3) after 24 h, (4) after 48 h, (5) after 7 days.

Initial tests for the preparation of such highly alkaline DADMAOH gels using the tetraallyl crosslinkers **1a–c** appeared only successful with TAAB (**1a**) and TAMPB (**1c**). The mixture with TAPB (**1b**) turned from colourless to yellow and dark orange in a short period of time without forming a gel and simultaneously developed a strong fish-like odour, indicating the release of amines. This is even more surprising, since poly(acrylate)s crosslinked with TAPB (**1b**) were found to be largely unaffected by boiling in NaOH solutions [14]. Therefore, control experiments regarding the hydrolytic stability of the crosslinkers **1a–c** and the previously used BIS were run by monitoring the ^1^H-NMR spectra in D_2_O containing 1 mol/L KOH over the course of seven days (Figure 2). The spectra in pure D_2_O were used as a reference.

**Figure 3:**
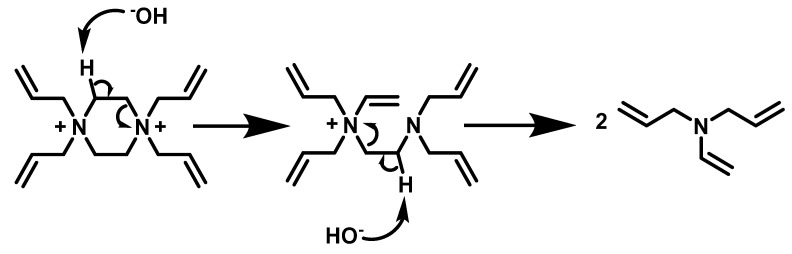
Potential beginning of the alkaline degradation of TAPB (**1b**) by Hofman-type elimination.

The control experiments showed no change in the appearance of the ^1^H-NMR spectra of TAAB (Figure 2a) and TAMPB (Figure 2c) over the period of 7 days. It can, thus, be concluded that these two are resistant to alkaline hydrolysis under these conditions. BIS, on the other hand, already shows (Figure 2d) the first signals of decomposition on the same day (Figure 2d(2)). Their intensity increases over time, while simultaneously the characteristic signals of BIS at 6.25 ppm, 5.83 ppm, and 4.75 ppm decrease and are completely lost after 48 h. In the case of TAPB (Figure 2b), signals of the crosslinker at 6.06 ppm, 5.86 ppm, 4.23 ppm, and 3.96 ppm could be identified on the same day in KOH/D_2_O, but these disappeared completely after 24 h (Figure 2b(3)). The susceptibility of TAPB to decomposition in alkaline media could be explained by a Hofmann-type elimination (Figure 3). The mechanism is particularly favoured by the proximity of the two positive charges on the piperazine ring, which creates tension within the ring due to the electrostatic repulsion and renders the hydrogen atoms α to the positive charges in **1b** more acidic. The latter can be indirectly observed by comparing the position of these H atoms (4.7 ppm, Figure 2b) to those in compound **1c** (3.7 ppm, Figure 2c), which are shifted to a higher field. The decrease of the signal intensity in the NMR is accompanied by the formation of a water-insoluble phase, which deposits a supernatant layer and appears to contain various decomposition products such as allylpiperazine, diallylvinylamine, and diallylamine, amongst others. An ^1^H-NMR spectrum of this in CDCl_3_ is given in Figure S1 (Supplementary Material).

As a result, TAPB (**1b**) is considered unsuitable as a crosslinker in the highly alkaline media and will therefore not be considered further. BIS, on the other hand, decomposes much more slowly and forms at least stable gels. BIS has previously been used to crosslink highly alkaline gels [8,9,10] and will therefore be used as reference for the allyl crosslinkers **1a** and **1c**.

**Figure 4:**
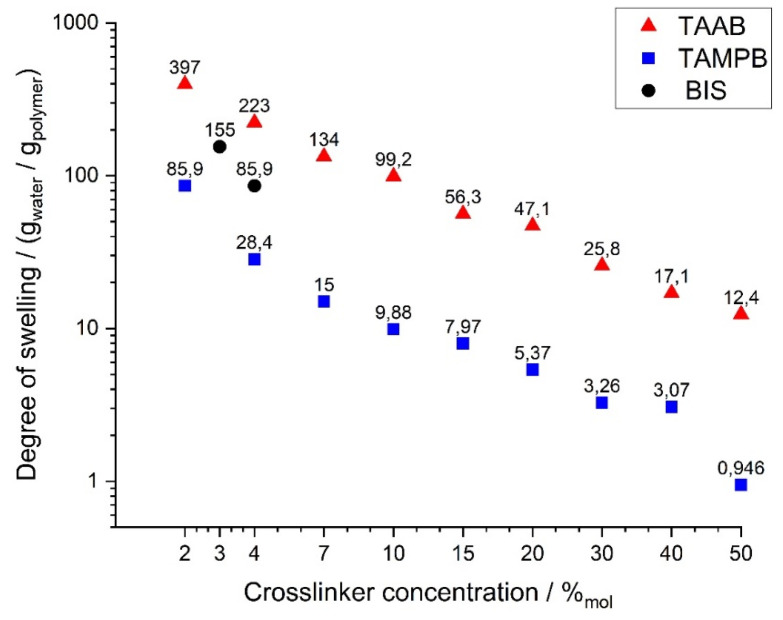
Swelling behaviour of poly(DADMAOH) gels with different crosslinkers as a function of the crosslinker concentration in bidistilled water. Reproducibility is approx. ±10–14%. Due to the logarithmic scale, the error bars are smaller than the symbols and are therefore omitted.

In order to find a suitable degree of crosslinking for the durability tests, the swelling properties of the gels were first determined as a function of the crosslinker content. For this purpose, DADMAOH hydrogels containing 2–50 mol% TAAB (**1a**) or TAMPB (**1c**) were prepared (Figure 4). In the case of BIS, the gels could only be prepared with 3 and 4 mol%, as amounts of less than 3 mol% did not result in stable gels, and 4 mol% is the solubility limit of BIS in this mixture. Technically, higher BIS/monomer ratios are possible at lower monomer concentrations, but such gels again exhibit poor mechanical stability. All gels were polymerized over a period of 3 weeks to ensure complete conversion. The comparatively long polymerization times are based on those previously observed for diallyldimethylammonium chloride gels [12]. 

Three trends can be observed from Figure 4: (i) as expected, the degree of swelling decreases for all gels with increasing crosslinker content, (ii) this seems to be less pronounced for TAAB (**1a**) than for TAMPB (**1c**), as the ratio of the degrees of swelling increases from 4.6 at 2% to 10 at 10% and to 13.1 at 50%, and (iii) TAAB (**1a**) consistently results in by far the highest degrees of swelling, with a measurable value of up to 397 g/g at a content of 2%. These values are comparable to common acrylate superabsorbent polymers [15,16]. BIS, on the other hand, is not only limited by the solubility but also by a seemingly much stronger decrease in the degree of swelling. Thus, the application range of gels with crosslinkers **1a**,**c** is much broader than that of the original BIS-DADMAOH system.

**Figure 5:**
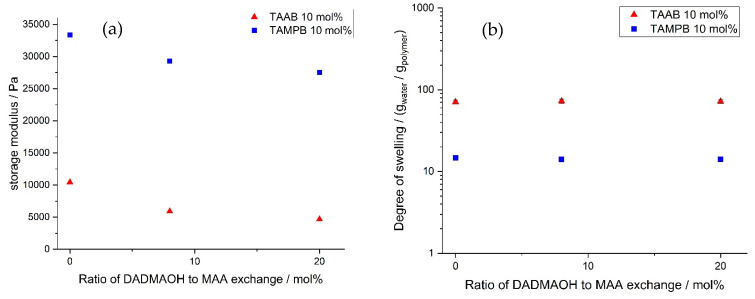
Storage modulus (**a**) and equilibrium degree of swelling (**b**) of gels crosslinked with 10 mol% TAAB (triangles) and TAMPB (squares), as a function of the molar fraction of methacrylamide. The storage modulus was determined at an amplitude of 1%, a frequency of 1 Hz, and a contact pressure of 5 N.

As stated above, DADMAOH crosslinked with 2% BIS does not form stable gels. To compensate this, methacrylamide (MAA) has previously been added as comonomer, which stabilizes the resulting gels due to the formation of hydrogen bonds and dipole-charge interactions [8]. As a side effect, the gels also become more pliable, which has a favourable effect on the processability. Although the addition of a comonomer to obtain coherent gels at low crosslinker contents is not necessary when using the tetraallyl compounds **1a**,**c**, it was interesting to test the effect of MAA on the present system in view of potential later applications. For this, the (chemical) crosslinker content was fixed at 10 mol% **1a**,**c,** and the MAA content was increased from 0 to 8 and then to 20% molar fraction; i.e., the ratio of crosslinker to the total amount of monomers was equal in all mixtures. After complete polymerization, the storage modulus and swelling properties of the resulting gels were analysed (Figure 5).

Figure 5a shows that for both TAAB (**1a**) and TAMPB (**1c**), the storage modulus decreases with increasing MAA content. This is in contrast to previous studies using 2 mol% BIS as a crosslinker, which noted a stiffening of the gels upon increasing MAA content [8]. The rate of decrease appears similar for both crosslinkers, despite the initial large difference in storage modulus (33.3 kPa for TAMPB, 10.4 kPa for TAAB). This leads to the assumption that the decrease is independent of the molecular structure. The decrease in storage moduli could, therefore, originate in a combination of two effects: gels crosslinked with tetraallyl compounds exhibit a homogeneous distribution of nodes, and at 10 mol% crosslinker, the gel appears to be too rigid for the weaker hydrogen bonds and dipole-charge interactions to be noticeable. On the other hand, the uncharged monomer reduces the charge density in the chains and reduces the electrostatic repulsion. This renders the chains more flexible. However, the reduction of the charge density does not seem to affect the swelling properties (Figure 5b). MAA was, therefore, used in the following experiments.

**Figure 6:**
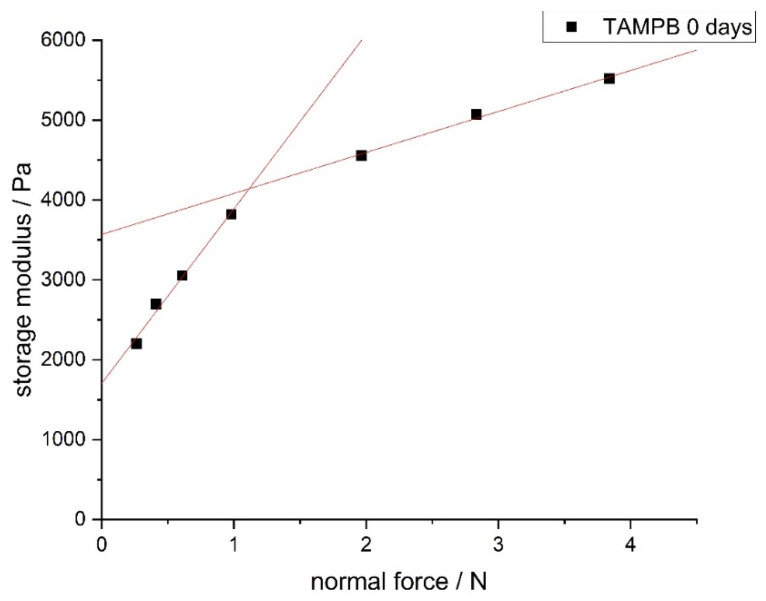
Storage modulus of a poly(DADMAOH) hydrogel with 2 mol% TAMPB and 8 mol% methacrylamid as a function of the applied normal force. The values were recorded at an amplitude of 1% and a frequency of 1 Hz 4 weeks after starting the polymerization.

For the rheological investigations, a crosslinker content of 2 mol% was selected for the TAMPB and TAAB gels and 4 mol% for the BIS gels due to the otherwise insufficient gel stability. In addition, 8 mol% methacrylamide based on DADMAOH was added to the polymerization solutions. Initially, all gels were cured in individual vials for 3 weeks and then 3–5 mm thick slices were cut from the centre of each gel block. The measurement errors due to the thickness variation of the gel slices are not significant here [17]. In order to be able to carry out the measurements reliably, the upper plate needs to contact the gel completely. This is not an issue in the case of very soft gels such as the ones crosslinked with BIS. For these heterogeneous gels, the dependence of storage modulus and normal force is not linear due to the macroporous structure, so measurements should be taken at low normal force [18]. Gels based on the tetraallyl crosslinkers **1a**,**c** appear firmer, despite the lower crosslinker content. Here, an additional pressure in the form of a normal force was needed to achieve full contact. Since it is known that this has a considerable influence on the determined moduli [17], the effect of the normal force on the present system was tested using a gel crosslinked with 2 mol% TAMPB (**1c**) at an amplitude of 1% (Figure 6).

Figure 6 shows that the observed storage modulus increases with increasing normal force, but with two different dependencies. At forces < 1 N, a very sharp increase can be seen, which changes to a significantly lower slope at forces > 1 N. From visual observation, the initial sharp increase can be addressed to the increasing contact area of the gels with the plates of the rheometer. In accordance with the literature, the subsequent region with lower slope is the result of the polymer chains in rigid gels being compressed, which results in macroscopic stiffening [17]. In order to remove the first effect, all samples crosslinked with TAAB (**1a**) and TAMPB (**1c**) were analysed using a normal force of 1 N. This was in agreement with the observations in the work of Karpushkin [18], where the values of approx. 0.1 to 1 N were reported. For significantly harder gels, it is necessary to use higher contact pressures, but these are determined by the same procedure in the later progress of the work.

**Figure 7:**
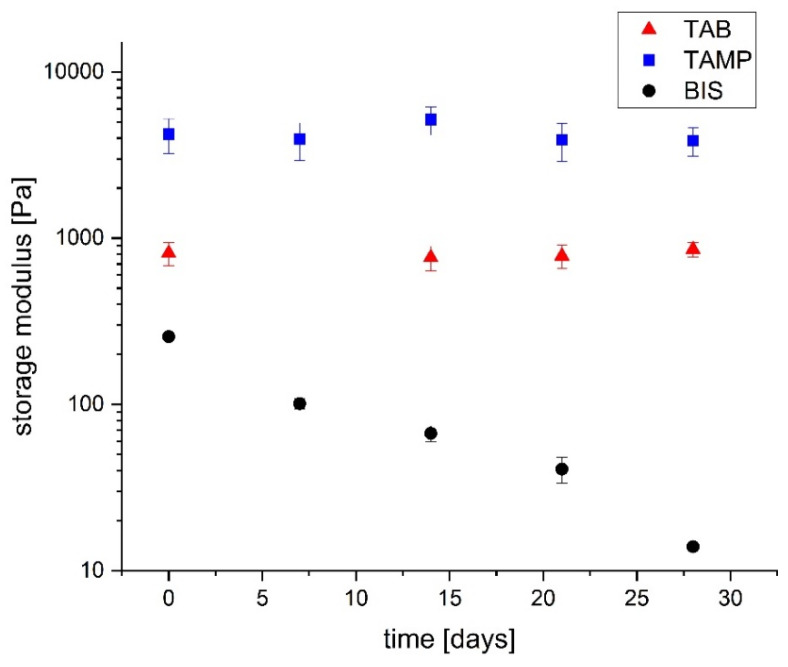
Change of the storage moduli of poly(DADMAOH-*co*-MAA)-hydrogels crosslinked with 2 mol% TAAB (triangles) or TAMPB (squares) and 4 mol% BIS (circles) at 60 °C.

To assess the susceptibility of the gels to alkaline hydrolysis, the polymerisation was allowed to continue for 3 weeks. The reference values (*t* = 0 in Figure 7) were determined at this point. The gels were then stored at room temperature and also at 60 °C and continuously monitored by analysing their rheological and swelling properties. At room temperature, a very small decrease in the storage modulus of BIS-crosslinked DADMAOH gels was observed, but not in those gels containing the tetraallyl-crosslinkers **1a**,**c**. The same applies to the degree of swelling at room temperature (Figures S2 and S3 in the Supplementary Materials). Therefore, the experiments were repeated at 60 °C to accelerate potential decomposition reactions (Figure 7).

Over the course of 28 days at 60 °C, the storage modulus of the BIS-crosslinked samples continuously decreased from 256 Pa to approx. 13 Pa. This enormous loss can also be observed haptically and visually, since after 28 days the gels were fluid. In contrast, the storage modulus of gels crosslinked with TAAB (**1a**) or TAMPB (**1c**) appeared to be constant. To corroborate this, a linear regression of the values in Figure 7 afforded a slope of −0.04 ± 0.0009 for gels crosslinked with BIS, while for the other two, the slope was 0 within the scatter of the measured values. On the molecular scale, the liquefaction can be explained by a degradation of the crosslinking points. This is a strong indication that gels crosslinked with **1a**,**c** are resistant to alkaline hydrolysis over the period of observation (28 d, 60 °C).

**Figure 8:**
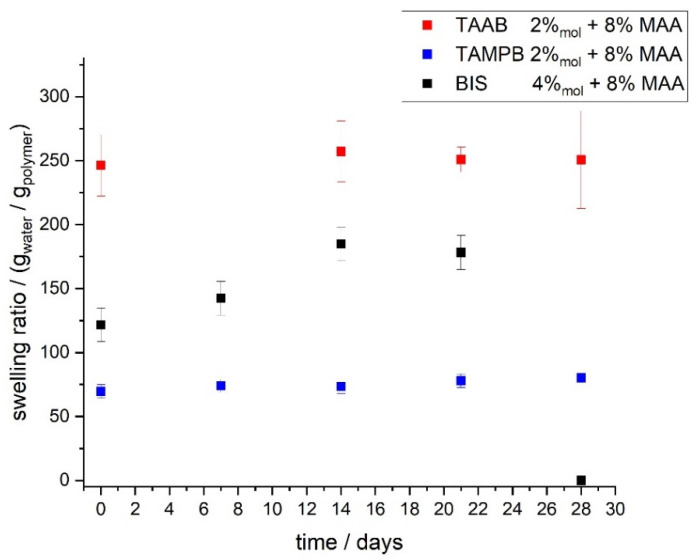
Equilibrium degree of swelling of poly(DADMAOH-*co*-MAA) hydrogels crosslinked with 2 mol% TAAB/2 mol% TAMPB/4 mol% BIS at different times after storage of the polymerized gels at 60 °C. All gels were lyophilized before the swelling tests.

In order to verify the above results, the swelling properties of the samples in Figure 7 were also determined (Figure 8).

Figure 8 shows that for TAAB-crosslinked gels, the degree of swelling is very much constant at approx. 250 g/g over the course of 28 days at 60 °C. The same applies to TAMPB-crosslinked gels, albeit with a lower degree of swelling of approx. 70 g/g. Since swelling mainly depends on the crosslinking density within the gels, it can be concluded that the crosslinking points persist in the highly alkaline environment. This confirms the findings in Figure 7. In contrast, gels crosslinked with BIS are again strongly affected, but despite the continuous decrease of the storage modulus shown in Figure 7, the degree of swelling increases at first from approx. 120 g/g to approx. 180 g/g. After 14 days, it decreases at an increasing rate and cannot be determined after 28 d since the sample is liquid. This also supports the decomposition of BIS already suspected in Figure 7. The initial increase is also, in our opinion, evidence for the beginning decomposition of the crosslinking points, since a lower crosslinking density gives—within certain limits—rise to higher degrees of swelling. The turning point over the course of the swelling curve is also initiated by parts of the gel flowing through the 90 µm wide meshes of the polyester teabag. As gels crosslinked with BIS have previously been shown to possess a non-uniform network structure [12], small amounts of hydrolysis might sever larger portions of polymer from the gel, which are then lost in the experiment.

## 3. Conclusions

The persistence of the present highly alkaline hydrogels depends on the nature of the crosslinker. The amide bonds in the commonly used *N*,*N*′-methylenebisacrylamide are subject to slow hydrolysis, which ultimately causes liquefication of the gels. Rather unexpectedly, *N*,*N*,*N*′,*N*′-tetraallylpiperazinium dibromide decomposes so quickly that alkaline gels do not form. The reason could be the proximity of the two positive charges in the six-membered ring, which renders the α-H atoms strongly acidic. This favours a Hofmann-type elimination. Gels crosslinked with tetraallylammonium bromide and *N*,*N*,*N*′,*N*′-tetraallyltrimethylene dipiperidine dibromide did not show any signs of decomposition after 28 d at 60 °C, which translates to at least 15 months at ambient temperature. This is sufficient for basically all relevant rehabilitation measures, such as realkalisation or chloride extraction. Both suitable tetraallyl crosslinkers allow the preparation of gels with a wide range of mechanical and swelling properties and are compatible with methacrylamide as comonomer, which renders the gels more pliable.

## 4. Materials and Methods

### 4.1. Materials

Diallyldimethylammonium chloride (65 wt % in H_2_O) and triallylamine (99%) were purchased from Sigma Aldrich, and piperazine and D_2_O (99.9%) were obtained from Merck KGaA (Darmstadt, Germany). Allyl bromide (99%), potassium carbonate (99%), methanol (99%), and 1,3-bis(4-piperidyl)propane (97+%) were purchased from Alfa Aesar (Kandel, Germany). The anion exchange-resin Lewatit Monoplus MP 800 was provided by Lanxess (Leverkusen, Germany). Chloroform (≥99%), acetone (≥99%), bidistilled water, potassium hydroxide, dichloromethane (≥99%), potassium persulfate, sodium metabisulphite, *N*,*N*′-methylenebisacrylamide and sodium hydroxide (97%) were obtained from VWR International GmbH (Darmstadt, Germany). All chemicals were used as received. Polyester filter bags with a maximum mesh size of 90 µm were purchased from Rosin Tech Products (Bethpage, NY, USA).

### 4.2. Synthesis of the Crosslinkers

Tetraallylammonium bromide **1a** (TAAB), *N*,*N*,*N*′,*N*′-tetraallylpiperazinium dibromide **1b** (TAPB) and *N*,*N*,*N*′,*N*′-tetraallyltrimethylenedipiperidine dibromide **1c** (TAMPB) were synthesised by using a previously published procedure [12]. Briefly, TAAB was prepared by reacting triallylamine with allyl bromide in acetone (80 °C, 72 h). The synthesis of **1b** was performed in two steps. First, piperazine was reacted with 2 eq. allyl bromide in H_2_O (20 °C, 48 h). The purification resulted in a yellow oily liquid. For the second step, the liquid was heated with 2.2 eq. allyl bromide in acetone (80 °C, 72 h) to give TAPB. Finally, the preparation of **1c** follows the two-step procedure outlined for TAPB. Trimethylenedipiperidine with 2 eq. allyl bromide afforded crude diallyltrimethylenedipiperidine (DAMP) as a brown oily liquid. In the second step, DAMP yielded TAMPB as a beige crystalline solid after recrystallization in methanol.

### 4.3. NMR Spectroscopy

^1^H-NMR spectra were recorded with 400 MHz on a Mercury 400 spectrometer (Varian, Palo Alto, CA, USA). Chemical shifts were calculated using the HDO signal at 4.64 ppm or the CDCl_3_ signal at 7.26 ppm as a reference. To check for potential alkaline hydrolysis, 25 mg of the crosslinkers were added to 1 mL of a solution containing 561.1 mg (0.01 mol) KOH in 10 mL D_2_O. ^1^H-NMR spectra were taken on the same day and after 24 h, 48 h, 72 h, and after 7 days. Spectra recorded in pure D_2_O were used as reference.

### 4.4. Preparation of Diallyldimethylammonium Hydroxide (DADMAOH) by Ion Exchange [8]

A total of 1125 g of the ion-exchange resin in the chloride form and 1.8 L of 1 M NaOH were placed in a chromatography column. The mixture was allowed to sit for 30 min before the column was drained and the resin washed with 1.2 L bidistilled water. For the exchange, 360 g of the 65 wt % commercial diallyldimethylammonium chloride (DADMAC) solution were diluted with 750 mL water and then slowly fed onto the column followed by 2.25 L bidistilled water. The combined solutions were adjusted to a concentration of 30 wt % by rotary evaporation. The dry weight was determined gravimetrically by freeze-drying 5 mL of the solution. The amount of chloride left in the product was determined by potentiometric titration and was for all samples less than 5 mol%.

### 4.5. Copolymerisation of Crosslinked DADMAOH Hydrogels

The method is described using 2 mol% of the TAAB (sample TAAB2) crosslinker as an example. Details on all compositions can be found in Table S1 in the Supporting Information. A mixture of 5 g of a DADMAOH solution (30 wt % in water, 10.5 mmol), 30 mg sodium disulfite (0.16 mmol), and 54 mg tetraallylammonium bromide (0.2 mmol, 2 mol% relative to the DADMAOH content) was stirred until the crosslinker had completely dissolved. Meanwhile, 60 mg KPS was dissolved separately in 1.5 mL H_2_O and then added to the monomer solution. All solutions were stirred for 15 min and then stored at room temperature for 3 weeks.

### 4.6. Swelling Experiments (Teabag Tests)

All tested gels were cut into small particles and lyophilized under reduced pressure. Approx. 100 mg of the dried hydrogels were weighed into polyester filter-bags and submerged in 0.5 L of bidistilled water at 22 °C. Every hour, the teabags were removed from the solution and carefully stripped off. To drain unabsorbed water, the samples were hung up for 5 min before the mass changes were measured gravimetrically. The reported maximum swelling ratios were determined by averaging the last three recorded values.

### 4.7. Preparation of Crosslinked DADMAOH-Co-Methacrylamide Hydrogels and Durability Test

The method is described using 2 mol% TAAB (sample TAAB2 + MAA) as an example. Details on all used compositions can be found in Table S2 in the Supporting Information. A mixture of 5 g of a DADMAOH solution (30 wt % in water, 10.5 mmol), 30 mg sodium disulfite (0.16 mmol), 54 mg tetraallylammonium bromide (0.2 mmol, 2 mol% related to the DADMAOH content), and 71.3 mg methacrylamide (0.8 mmol, 8 mol%) was stirred until the crosslinker had completely dissolved. Meanwhile, 60 mg KPS was dissolved separately in 1.5 mL H_2_O and then added to the monomer solution. The solutions were stirred for 15 min and then sealed with parafilm to avoid drying effects. After storing the samples at room temperature for 3 weeks, half of the samples were placed in a Memmert UN55 drying oven at 60 °C and kept there over a period of 4 weeks. The samples were evaluated by rheological experiments according to Section 4.8 or by swelling experiments according to Section 4.6 after lyophilisation. For that, the glass vial of a sample was broken, and the gel was carefully removed. A 3–5 mm thick gel slice was then cut from the centre of the gel cylinder. Care was taken to ensure that the thickness of the slice remained the same over the entire surface. The first (reference) measurement was made after the initial 3 weeks at RT. Further measurements were made after an additional 6 weeks at RT, as well as after 7/14/21 and 28 days of storage at 60 °C. At the corresponding time points, the sample vials were carefully shattered, and the bulk gel was removed from the glass fragments.

### 4.8. Rheology

The rheological data were recorded on an Anton Paar Modular Compact Rheometer 102. A plate–plate geometry with a diameter of 25 mm and made of stainless steel was used. All samples were measured at 20 °C. The gap distance was selected depending on the applied normal force, which was set between 0 and 5 N depending on the experiment. For each measurement, an amplitude sweep was performed, with an angular frequency of 1 Hz and an increasing amplitude of 0.01–100% over 25 measurement points.

### 4.9. Ratio Exchange Tests of Crosslinked DADMAOH-Co-Methacrylamide Hydrogels

Three separate solutions were prepared from the 30% DADMAOH solution obtained in Section 4.4, in which equimolar 0/8/20 mol% DADMAOH monomers were exchanged by dilution with bidistilled water and the subsequent addition of methacrylamide. For each of the three solutions, 3 reaction mixtures for each of the crosslinkers were prepared as follows. For each sample, a mixture of 5 g of one of the above three DADMAOH-MAA-solutions (10.5 mmol), 30 mg sodium disulfite (0.16 mmol), 270 mg TAAB, or 557.5 mg TAMPB (0.2 mmol, 10 mol% related to the DADMAOH-MAA content), was stirred until the crosslinker had completely dissolved. Meanwhile, 60 mg KPS was dissolved separately in 1.5 mL H_2_O and then added to the monomer solution. All solutions were stirred for 15 min and then sealed with parafilm to avoid drying effects. All samples were stored at room temperature for 3 weeks and then analyzed for storage moduli and swelling ratios according to the above procedures.

## Data Availability

Data are available upon reasonable request.

## Supplementary Materials

The following are available online at https://www.mdpi.com/article/10.3390/gels8100669/s1, Figure S1: ^1^H-NMR spectrum in CDCl_3_ of the supernatant was performed during the decomposition of **1b** in 1 m KOH; Figure S2: Storage moduli of poly(DADMAOH-*co*-MAA) hydrogels crosslinked with 2 mol% TAAB, 2 mol% TAMPB, and 4 mol% BIS at different times after storage of the polymerized gels at room temperature; Figure S3: Swelling ratios of poly(DADMAOH-*co*-MAA)-hydrogels crosslinked with 2%_mol_ TAAB/2%_mol_ TAMPB/4%_mol_ BIS at different times after storage of the polymerized gels at room temperature; Table S1: Details on the sample composition used to prepare crosslinked DADMAOH hydrogels; Table S2: Details on the sample composition used to prepare crosslinked DADMAOH-*co*-MAA hydrogels.
