# Supplementary material for: Hydrolytic Stability of Crosslinked, Highly Alkaline Diallyldimethylammonium Hydroxide Hydrogels
Source: Gels. 2022 Oct 19;8(10):669. doi: 10.3390/gels8100669 (PMC9601492; doi:10.3390/gels8100669)
Supplement: Supplementary file 1 [file gels-08-00669-s001.zip › gels-1954039-supplementary.pdf]

Supplementary Materials:

To

Hydrolytic stability of crosslinked, highly alkaline diallyldimethylammonium hydroxide hydrogels

Tim B. Mrohs [1] and Oliver Weichold[1,*]

[1] Institute of Building Materials Research, RWTH Aachen University, Schinkelstraße 3, 52062 Aachen, Germany.

* Correspondence: weichold@ibac.rwth-aachen.de

Table S1: Details on the sample composition used to prepare crosslinked DADMAOH hydrogels.

	Crosslinker			
**Sample**	**Type**	**ratio / mol-%**	***n* / mmol**	***m* / mg**
BIS3	BIS	3	0,3	48
BIS4	BIS	4	0,4	64
TAAB2	TAAB	2	0,2	54
TAAB4	TAAB	4	0,4	108
TAAB7	TAAB	7	0,7	189
TAAB10	TAAB	10	1	270
TAAB15	TAAB	15	1,5	405
TAAB20	TAAB	20	2	540
TAAB30	TAAB	30	3	810
TAAB40	TAAB	40	4	1080
TAAB50	TAAB	50	5	1350
TAPB2	TAPB	2	0,2	85,5
TAMPB2	TAMPB	2	0,2	111,5
TAMPB4	TAMPB	4	0,4	223
TAMPB7	TAMPB	7	0,7	390
TAMPB10	TAMPB	10	1	557
TAMPB15	TAMPB	15	1,5	835
TAMPB20	TAMPB	20	2	1114
TAMPB30	TAMPB	30	3	1671
TAMPB40	TAMPB	40	4	2228
TAMPB50	TAMPB	50	5	2785

Table S2: Details on the sample composition used to prepare crosslinked DADMAOH-*co*-MAA hydrogels**.**

	Crosslinker				Methacrylamide	
**Sample**	**Type**	**ratio / mol-%**	***n* / mmol**	***m* / mg**	***n* / mmol**	***m* / mg**
BIS4 + MAA	BIS	4	0,4	64	0,8	71,3
TAAB2 + MAA	TAAB	2	0,2	54	0,8	71,3
TAMPB2 + MAA	TAMPB	2	0,2	111,5	0,8	71,3

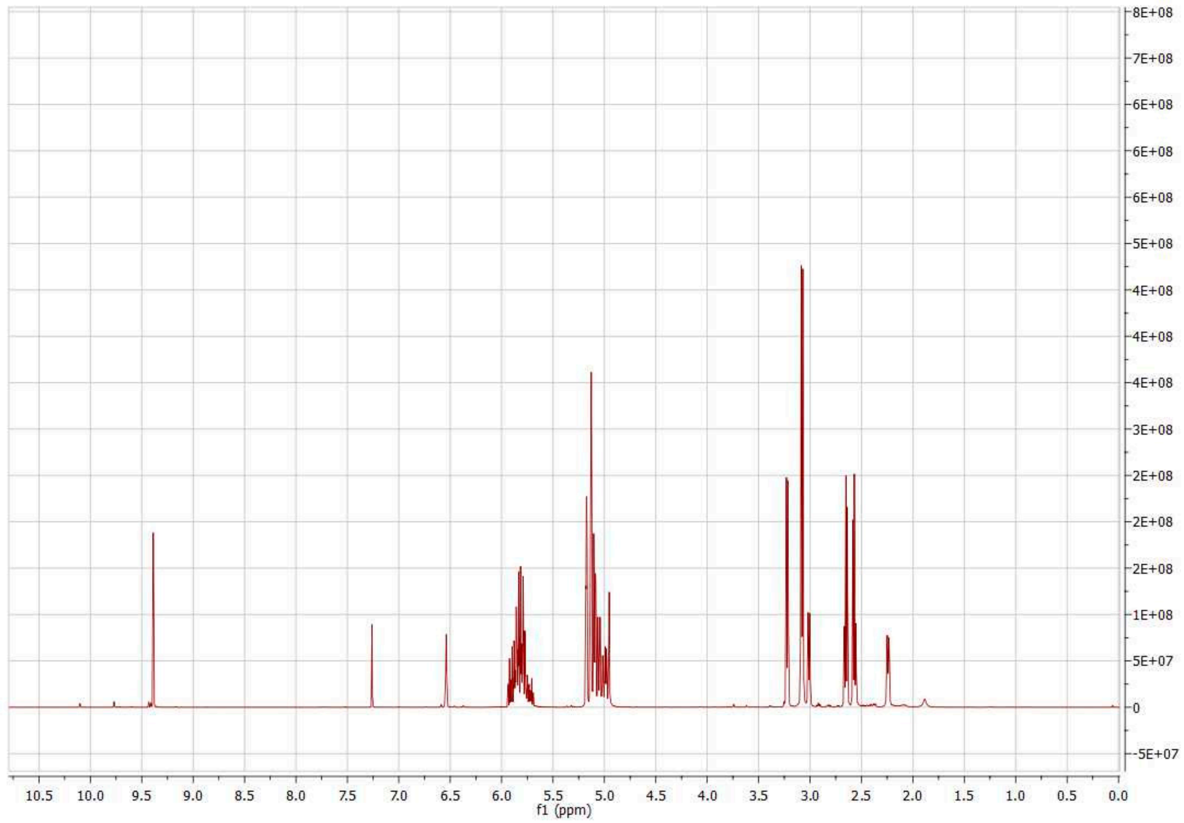

Figure S1. TAPB in 1 M KOH/D_2O-solution after 168 hours incubation. A supernatant has formed, from which a ^{1}H-NMR-Spectrum was performed in $CDCl_3$.

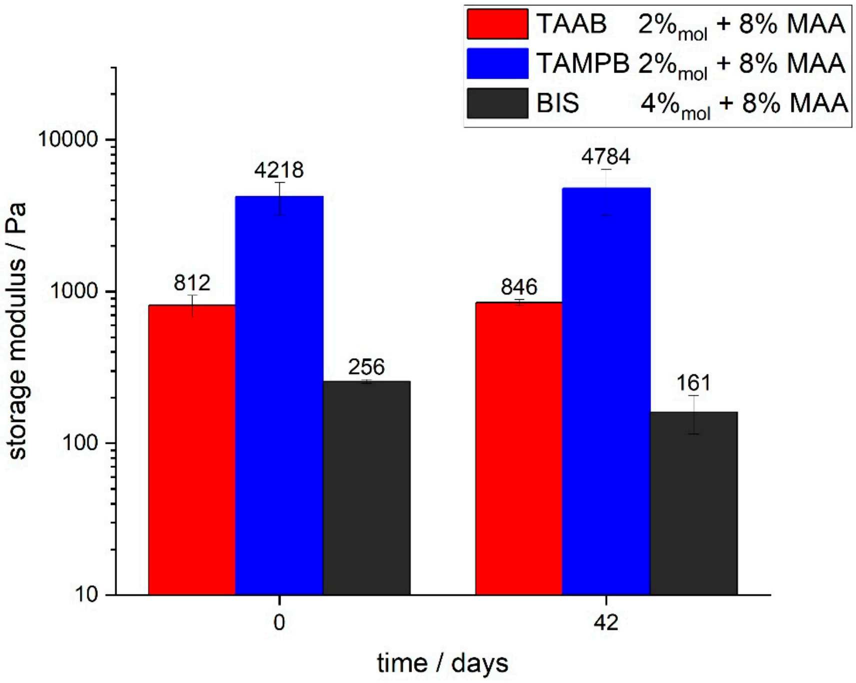

Figure S2. Storage moduli of poly(DADMAOH-*co*-MAA)- hydrogels crosslinked with 2 mol% TAAB, 2 mol% TAMPB, and 4 mol% BIS over the course of 6 weeks at room temperature.

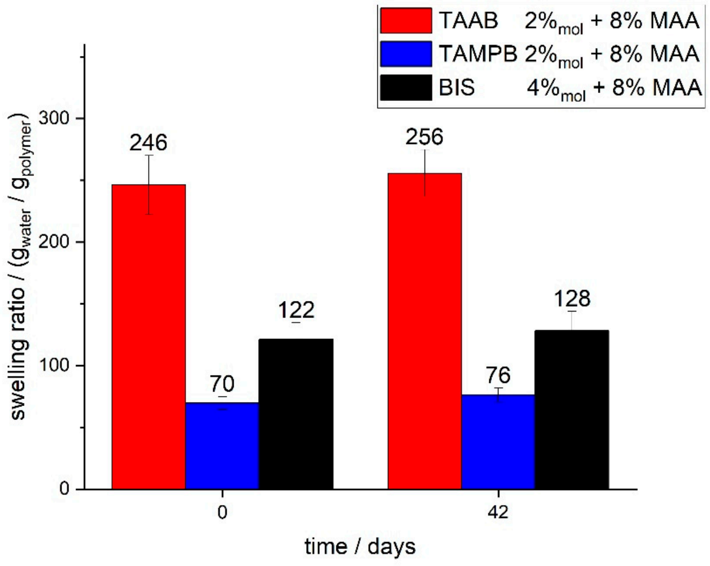

Figure S3. Swelling ratios of poly(DADMAOH-*co*-MAA)- hydrogels crosslinked with 2 mol% TAAB, / 2 mol% TAMPB, and 4 mol% BIS over the course of 6 weeks at room temperature. All gels were lyophilized prior to the swelling tests.
